# Navigating academic expectations and social integration: a moderated mediation analysis of cultural adaptation, peer support, and well-being among Chinese international students

**DOI:** 10.3389/fpsyg.2025.1633773

**Published:** 2025-11-21

**Authors:** Jincheng Xu, Wenyu Chai

**Affiliations:** School of Graduate Studies, Lingnan University, Hong Kong, Hong Kong SAR, China

**Keywords:** academic expectations, cultural adaptation, social integration, peer support, student well-being, acculturative stress

## Abstract

International students often encounter significant academic and cultural challenges that can adversely affect their psychological well-being. This study examines the association between academic expectations and well-being of Chinese international students, with cultural adaptation and social integration modeled as parallel mediators, and peer support as a moderator. A cross-sectional survey was conducted among 456 Chinese international students enrolled in universities. The findings reveal that academic expectations have a direct negative effect on student well-being and also exert significant indirect effects through both cultural adaptation and social integration. Moreover, peer support moderates the negative relationship between academic expectations and both mediators, thereby reducing their adverse association with well-being. The moderated mediation analysis confirms that high levels of peer support buffer the detrimental effects of academic pressure by enhancing students’ adaptive and integrative capacities. These results underline the importance of fostering peer-based support systems and culturally responsive interventions in promoting international student well-being. The study contributes to the growing body of research on acculturative stress and offers practical implications for student affairs professionals and institutional policymakers.

## Introduction

The global rise in international education has reshaped the landscape of higher learning, with students increasingly crossing borders in pursuit of academic and personal growth. In particular, Chinese international students represent one of the largest and most mobile student groups globally, contributing significantly to campus diversity and internationalization efforts (ICEF Monitor, 2024; UNESCO Institute for Statistics, 2024). Despite these benefits, their experiences often involve substantial adjustment difficulties, including unfamiliar pedagogical practices, language barriers, and social isolation, all of which can negatively influence their psychological well-being (Zhao and Schartner, 2024). Prior studies have largely focused on academic performance and cultural diversity, but fewer have explored the stress-related psychological challenges these students face in host academic environments (Lam and Zhou, 2022; Markey et al., 2023; Wang et al., 2023).

In addition, academic demands in unfamiliar educational contexts can become a key source of psychological stress. Several scholars argue that international students frequently encounter heightened academic expectations, which include pressure to achieve high grades, meet rigid performance standards, and satisfy family or financial obligations tied to academic success (Huang et al., 2024; Mulyadi et al., 2024). When students perceive a mismatch between these expectations and their coping resources, it often leads to academic stress, performance anxiety, and even depressive symptoms (Bai et al., 2024). While the academic adjustment of international students has been widely examined, its psychological implications—particularly its association with well-being—have not been sufficiently addressed in integrated models.

Furthermore, scholars have increasingly emphasized that cultural adaptation is not simply a matter of surface-level familiarity but a deeper, ongoing process of psychological and behavioral adaptation (Campos et al., 2022). In this regard, researchers posit that cultural adaptation is a vital element in acculturation, involving individuals’ efforts to understand and integrate the norms, communication styles, and institutional practices of the host society (Aloka et al., 2023; Soares et al., 2021). Successful cultural adaptation has been associated with lower levels of emotional distress and greater academic satisfaction among international students (Yılmaz and Temizkan, 2022). However, existing literature has predominantly viewed cultural adaptation as an outcome variable, rather than exploring its function as a mediator that may explain how academic pressures influence psychological well-being (Campos et al., 2022; Gong et al., 2021).

Previous studies have also acknowledged the importance of social ties and interpersonal belonging in enhancing international students’ emotional stability. For instance, social integration, which refers to students’ perceived sense of belonging and participation in the host university’s social community, plays a protective role in mitigating feelings of loneliness and alienation (Bianchi and Martini, 2023). A growing body of research links social integration to improved psychological adjustment, academic satisfaction, and reduced levels of stress and homesickness (De Bruyn and Van Eekert, 2023; Franz and Paetsch, 2023). Yet, limited empirical work has examined how social integration functions as a psychological mechanism within stress-related models, particularly in culturally diverse student populations outside Western contexts.

In addition to the mediating mechanism, a supportive peer network has been identified as a crucial external resource that helps international students navigate academic and cultural challenges. Peer support encompasses emotional encouragement, academic assistance, and companionship, which collectively enhance students’ sense of competence and belonging (Fraser et al., 2024). Prior studies indicate that students with strong peer relationships report fewer symptoms of anxiety and demonstrate higher levels of adaptive functioning in multicultural settings (Li et al., 2025; Xu et al., 2024). Despite its significance, the moderating role of peer support in shaping the link between academic stress and psychological adaptation remains underexplored, especially in non-Western or intra-Asian student mobility contexts.

The conceptual framework for this study is informed by Acculturative Stress Theory, which posits that individuals experience psychological strain when attempting to adapt to a new cultural environment (Berry, 1997). Within this framework, stress-inducing factors such as academic expectations may undermine well-being, yet these effects can be mediated or moderated by factors like cultural adaptation, social integration, and peer support. While Acculturative Stress Theory has been widely applied in studies of international student adjustment, recent meta-analytical findings suggest that few empirical investigations have extended this framework through moderated mediation designs that capture both direct and conditional indirect effects on student well-being (Soufi Amlashi et al., 2024).

Taken together, the present study seeks to examine the relationship between academic expectations and student well-being among Chinese international students, with cultural adaptation and social integration as mediating variables and peer support as a moderator. By integrating these variables into a single framework grounded in acculturative stress theory, the study contributes to a deeper understanding of the psychological processes influencing student adjustment. The findings are expected to inform institutional practices and student support policies aimed at enhancing well-being and promoting inclusive, culturally responsive educational environments.

### Research objective

Based on the above discussion, the research objectives of this study are as follows:

- To examine the association between academic expectations and student well-being.
- To investigate the mediating effect of cultural adaptation on the relationship between academic expectations and student well-being.
- To explore the mediating effect of social integration on the relationship between academic expectations and student well-being.
- To study the moderating role of peer support on the relationship between academic expectations and cultural adaptation.
- To study the moderating role of peer support on the relationship between academic expectations and social integration.
- To examine the moderated mediation effect of peer support in the indirect relationship between academic expectations and student well-being through cultural adaptation and social integration.

### Hypotheses development and conceptual model

#### Academic expectations and student’s well-being

Academic expectations refer to the perceived academic demands placed on students, including the pressure to achieve high grades, manage unfamiliar workloads, and meet the expectations of parents, faculty, or scholarship bodies (Aloka et al., 2023). For international students, these expectations often become more stressful due to differences in academic cultures, language barriers, and limited familiarity with host institutions’ teaching methods (Campos et al., 2022). When such demands exceed a student’s coping resources, they can lead to chronic stress, academic burnout, and deteriorating psychological health (Cassidy and Boulos, 2023). Student well-being, in contrast, encompasses psychological, emotional, and social aspects of students’ mental health, including feelings of life satisfaction, emotional stability, and a positive academic experience (Van de Velde et al., 2021).

According to Acculturative Stress Theory (Berry, 1997), international students experience psychological stress when they encounter difficulties adjusting to the cultural and academic expectations of a host environment. These stressors arise when there is a misfit between students’ internal coping resources and external academic demands, resulting in heightened psychological strain (Ripani, 2024). Academic expectations, in this context, include students’ perceptions of workload, performance standards, competitive environments, and the pressure to fulfill family or institutional obligations (Liu et al., 2022). When international students perceive these expectations as overwhelming or unmanageable, the likelihood of anxiety, burnout, and emotional distress significantly increases (Dwumah Manu et al., 2023). This misalignment may be intensified by language barriers, lack of familiarity with assessment styles, or limited social and institutional support, which collectively amplify their vulnerability to psychological disruption (Luz and Thomas, 2023).

Numerous studies have demonstrated that elevated academic stress correlates with reduced levels of mental well-being, lower academic satisfaction, and greater psychological disengagement (Guszkowska and Dąbrowska-Zimakowska, 2022). International students facing unmet academic expectations often experience feelings of helplessness, inadequacy, and diminished self-worth, which further erode their emotional stability (Ripani, 2024). The compounded effect of academic overload and adjustment strain can lead to severe outcomes, including depressive symptoms, reduced academic motivation, and social withdrawal (Li et al., 2025). Given the theoretical and empirical foundations supporting the negative consequences of academic stress on mental health, the following hypothesis is proposed:

*H1:* Academic expectations are negatively associated with student well-being.

#### Mediating role of cultural adaptation

Previous studies have emphasized that successful adjustment to a host culture plays a pivotal role in determining the psychological outcomes of international students. Cultural adaptation refers to the process through which individuals acquire the knowledge, skills, and behaviors necessary to function effectively in a new cultural environment, including adapting to academic systems, communication styles, and societal expectations (Campos et al., 2022). It involves not only surface-level adjustments but also deeper cognitive and emotional shifts that help students manage day-to-day experiences more competently (Yılmaz and Temizkan, 2022). Research has consistently shown that individuals who adapt well culturally experience lower levels of psychological distress, increased self-efficacy, and a stronger sense of personal control in academic and social domains (Gong et al., 2021).

Cultural adaptation has been found to positively associate student well-being with reduced feelings of alienation, uncertainty, and academic frustration, thereby fostering emotional stability and life satisfaction (Olagunju et al., 2024). Studies suggest that culturally adapted students are more likely to engage in classroom interactions, seek help when needed, and form meaningful relationships, which in turn enhance their psychological functioning (Zhang et al., 2022). Conversely, poor adaptation has been associated with increased vulnerability to stress, depressive symptoms, and academic disengagement (Aloka et al., 2023). Within the framework of Acculturative Stress Theory (Berry, 1997), cultural adaptation serves as a buffer by transforming environmental challenges into manageable experiences through cognitive and behavioral adjustments.

In the context of academic expectations, students who are better adapted to the host culture may perceive academic demands as more comprehensible and manageable, reducing the stress these demands typically generate (Liu et al., 2022; Olagunju et al., 2024). This suggests that cultural adaptation may function as a psychological mechanism through which academic expectations are associated with well-being. Empirical evidence supports this mediating pathway, showing that adaptation skills can mitigate the negative effects of academic stress and facilitate more effective coping strategies (Wang et al., 2021). Therefore, it is hypothesized that:

*H2a:* Cultural adaptation mediates the relationship between academic expectations and student well-being.

#### Mediating role of social integration

In addition to cultural adaptation, social integration has been identified as a crucial determinant of international students’ psychological well-being. Social integration refers to the extent to which students feel connected to and accepted by the social and academic communities within the host institution, including participation in peer interactions, engagement in campus activities, and perceptions of belonging (Bianchi and Martini, 2023). It reflects not only structural involvement but also the emotional and psychological bond that students develop with their educational and social environment. Previous research suggests that higher levels of social integration are associated with improved psychological outcomes such as reduced loneliness, increased life satisfaction, and enhanced emotional resilience (De Bruyn and Van Eekert, 2023).

A socially integrated student is more likely to benefit from informal academic support, peer encouragement, and emotional companionship, which collectively promote psychological stability (Johnson and Goldman, 2023). Conversely, students who feel socially excluded or disconnected often experience elevated levels of stress, homesickness, and anxiety, which can compromise their academic functioning and overall well-being (Baert, 2023). Within the framework of Acculturative Stress Theory (Berry, 1997), social integration acts as a resource that buffers the negative psychological effects of adaptation-related stressors by fostering interpersonal connectedness and emotional support.

In the context of academic expectations, students who are socially well-integrated may perceive academic challenges as more manageable due to the encouragement, shared experiences, and practical help they receive from peers (Tlalajoe-Mokhatla, 2024). These social ties reduce the psychological burden imposed by high academic demands, thereby supporting the idea that social integration mediates the relationship between academic stress and well-being. Prior studies support this indirect pathway, demonstrating that students who report higher social integration experience less academic anxiety and greater satisfaction with their overall university experience (Jia et al., 2022; Johnson and Goldman, 2023). Based on these insights, the following hypothesis is proposed:

*H2b:* Social integration mediates the relationship between academic expectations and student well-being.

#### Moderating role of peer support

While individual adaptation and social inclusion are critical for international student adjustment, researchers increasingly emphasize the role of interpersonal resources in shaping how stress is experienced and managed. Peer support, in particular, has emerged as a salient moderating factor in models of psychological well-being, as it encompasses emotional reassurance, academic guidance, and companionship provided by fellow students (Fraser et al., 2024). The presence of supportive peer relationships contributes to a safe and affirming environment, allowing students to express concerns, receive encouragement, and share coping strategies during challenging academic or cultural transitions (Li et al., 2025). Within such supportive peer networks, students are more likely to navigate unfamiliar norms, overcome language barriers, and develop confidence in their academic and social roles (Xu et al., 2024).

The theoretical foundation for this argument is embedded in the buffering hypothesis of social support, which posits that interpersonal support can weaken or “buffer” the negative relationship between stressors and psychological outcomes (Ye et al., 2021). In the context of acculturative stress theory (Berry, 1997), peer support functions as a cultural and emotional scaffold that softens the stress-inducing effects of adaptation pressures. When academic expectations are high, peer support can serve as a protective factor that facilitates interpretation of those demands as less threatening or overwhelming (Bradley et al., 2021). Research has shown that students who feel emotionally and socially supported by peers report lower levels of stress, fewer depressive symptoms, and greater overall well-being, particularly in multicultural educational settings (Seery et al., 2021). These effects are especially pronounced among international students who rely heavily on peer-based interaction due to distance from familial support systems (Permatasari et al., 2021).

Specifically, peer support may moderate the relationship between academic expectations and students’ capacity for cultural adaptation and social integration. When peer support is high, the negative relationship between academic stress and cultural adaptation may be reduced, as peers can guide newcomers through institutional expectations, help interpret unwritten norms, and model culturally appropriate behaviors (De Clercq et al., 2021). Similarly, peer support may buffer the negative relationship between academic expectations and social integration by promoting inclusion, fostering emotional connection, and facilitating access to informal social networks (Li et al., 2025). These moderating pathways are supported by empirical findings showing that students with strong peer support are more resilient and socially active, even under high academic pressure (Edwards et al., 2022; Putri, 2024).

Taken together, the study proposes that:

*H3:* Peer support moderates the relationship between academic expectations and cultural adaptation, such that the negative effect is weaker at higher levels of peer support.

*H4:* Peer support moderates the relationship between academic expectations and social integration, such that the negative effect is weaker at higher levels of peer support.

#### Moderated mediation model

Building upon the mediating roles of cultural adaptation and social integration, and the moderating role of peer support, this study proposes a moderated mediation model to capture the complexity of international student adjustment under academic stress. A moderated mediation model explains how and under what conditions an indirect relationship occurs (Preacher et al., 2007). In this study, academic expectations are linked with student well-being indirectly through two mediators—cultural adaptation and social integration—and these indirect effects are conditional on levels of peer support. This model provides a more comprehensive account of how international students experience and process academic challenges, emphasizing both internal adaptive processes and external contextual resources.

According to Acculturative Stress Theory (Berry, 1997), the psychological effects of stress-inducing factors such as academic expectations are filtered through mechanisms like adaptation and integration. However, these mechanisms do not function uniformly across individuals; they are shaped by the surrounding social environment, particularly the availability of interpersonal support. As posited by the buffering hypothesis of social support (Putri, 2024), peer support can alter the strength and direction of stress-related outcomes. When peer support is high, students are more likely to adapt to cultural demands and integrate socially despite elevated academic expectations. This means that the indirect, negative effects of academic pressure on well-being may be significantly diminished—or even rendered non-significant—when peer support is present at sufficient levels.

Empirical evidence supports the presence of such conditional indirect effects in international student populations. Shahid and Shabbir (2021) and Kaur and Sharma (2022) observed that interpersonal support systems enhanced both cultural and social adjustment outcomes, thereby protecting students’ mental health under high academic pressure. Similarly, Kuo et al. (2021) and Ayhan and Bilgin (2024) found that peer support not only mitigated acculturative stress but also moderated the link between stressors and adaptation-related outcomes. When integrated into a moderated mediation framework, these findings suggest that peer support relate the entire indirect path from academic expectations with well-being through both adaptation and integration channels. Thus, the strength of the mediating effect depends on the level of peer support a student receives.

By examining these conditional indirect effects, the present study extends prior models of international student adjustment and highlights how institutional interventions can be tailored to strengthen peer-based support systems. Based on the theoretical and empirical rationale, the following hypotheses are proposed:

*H5a:* Peer support moderates the indirect effect of academic expectations on student well-being through cultural adaptation, such that the mediated effect is weaker at higher levels of peer support.

*H5b:* Peer support moderates the indirect effect of academic expectations on student well-being through social integration, such that the mediated effect is weaker at higher levels of peer support.

**Figure 1:**
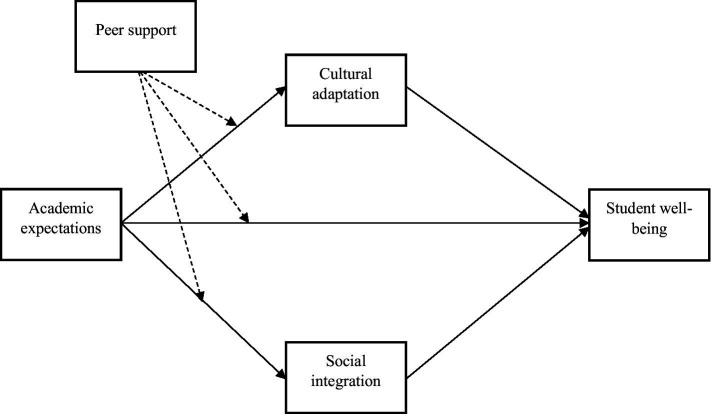
Conceptual model [dashed lines represent moderation paths (academic expectations × peer support)].

The conceptual model of the study is presented in Figure 1.

### Research methodology

This study adopted a cross-sectional research design, which is suitable for examining relationships among psychological and behavioral constructs at a single point in time. Given the nature of the variables—academic expectations, cultural adaptation, social integration, peer support, and well-being—a cross-sectional approach allows for efficient data capture without the logistical constraints of longitudinal tracking, especially in a population of mobile and time-constrained international students.

The sampling technique employed was purposive sampling, targeting Chinese international students enrolled at universities in the host country. This technique is justified by the study’s focus on a specific subpopulation with unique acculturative and academic experiences. Purposive sampling ensured that only students who met the inclusion criteria—being of Chinese origin, currently enrolled in higher education, and having studied for at least one semester—were invited to participate, thereby increasing the relevance and validity of the responses.

Data were collected using an online survey distributed through institutional academic advisors, international student support offices, and university WeChat groups. The questionnaire was available in both English and Mandarin to enhance comprehension and participation. A total of 650 questionnaires were distributed electronically. Of these, 487 were returned, and after screening for incomplete or patterned responses, 456 valid responses were retained for analysis. This yields a response rate of approximately 70.2%, which is considered high for online surveys in cross-cultural academic settings.

To improve participation and response quality, a follow-up reminder was sent one week after the initial distribution. The data collection process spanned eight weeks, from February 2025 to April 2025, ensuring sufficient time for participation while maintaining temporal consistency across responses.

#### Demographic profile

The final sample consisted of 456 Chinese international students enrolled in undergraduate and postgraduate programs across various universities. Of the respondents, 61.2% were female and 38.8% were male. The majority of participants (73.5%) were between the ages of 18 and 25 years. In terms of academic level, 64.9% were pursuing undergraduate degrees while 35.1% were enrolled in postgraduate programs. Most participants had been residing in the host country for more than six months, and 82.4% reported having at least moderate proficiency in the local language, indicating an adequate level of cultural exposure for valid responses related to adaptation and integration (Appendix A).

#### Common method biasness

To address the concern of common method bias (CMB), both procedural and statistical remedies were employed. Procedurally, anonymity and confidentiality were assured to reduce social desirability and evaluation apprehension. The items were carefully constructed and spread across different sections of the questionnaire to reduce item priming effects and response patterning. Additionally, the survey was presented bilingually (English and Mandarin) to minimize comprehension-related biases.

Statistically, Harman’s single-factor test was conducted, in which all items from the main constructs were loaded into an exploratory factor analysis. The results showed that the first unrotated factor accounted for only 28.4% of the total variance, which is well below the 50% threshold, suggesting that common method bias was not a serious concern in this study.

However, given the limitations of Harman’s test, we further performed a Confirmatory Factor Analysis (CFA) single-factor model, which demonstrated poor fit compared with the proposed measurement model (χ^2^/df = 7.12, CFI = 0.62, TLI = 0.58, RMSEA = 0.11, SRMR = 0.09), confirming that CMB is not a critical concern.

In addition, full-collinearity Variance Inflation Factors (VIFs) were computed for all latent constructs (ranging from 1.57 to 2.22), all well below the conservative threshold of 3.3 (Kock, 2015), providing further evidence that common method variance is unlikely to bias the results. A marker-variable approach was also assessed and yielded nonsignificant correlations with the main constructs, supporting the robustness of the findings. The summary of these tests is presented in Appendix B.

### Measures

All constructs in the study were measured using multi-item scales adapted from previously validated instruments. A 5-point Likert scale was used for all items, ranging from 1 (Strongly Disagree) to 5 (Strongly Agree), to ensure consistency and ease of understanding for respondents. The scales were slightly modified for contextual relevance to Chinese international students, and a back-translation procedure was employed to ensure linguistic and conceptual equivalence between the English and Mandarin versions (Brislin, 1986), while preserving their original psychometric properties.

Academic expectations were measured using a 4-item scale adapted from Ang and Huan (2006), focusing on perceived academic pressure and fear of failure. Cultural adaptation was assessed using 6 items adapted from the Sociocultural Adaptation Scale (SCAS) developed by Ward and Kennedy (1999), capturing students’ adjustment to academic and social norms in the host culture. Social integration was measured using 3 items adapted from the work of Tinto (2012) and later studies on international student engagement, focusing on belongingness and participation. Peer support was assessed using 5 items adapted from the Multidimensional Scale of Perceived Social Support (MSPSS) – Friends Subscale (Zimet et al., 1988), reflecting the emotional and instrumental support received from peers. Well-being was measured using 5 items adapted from the World Health Organization’s WHO-5 Well-Being Index (World Health Organization, 1998), which evaluates subjective mental and emotional well-being. These adapted measures ensured theoretical alignment with the study’s conceptual model and were tested for reliability and validity during the measurement model assessment phase.

#### Analytical strategy

The study employed Partial Least Squares Structural Equation Modeling (PLS-SEM) using SmartPLS 4.0.8 to test the hypothesized relationships among variables. PLS-SEM was selected because the proposed framework integrates multiple mediation and moderation effects, emphasizing prediction and explained variance rather than overall model fit. According to Hair et al. (2019), it performs robustly under non-normal data conditions and is particularly suitable for complex conditional-process models. Given the sample size (*N* = 456) and reflective constructs, this approach ensures consistent and efficient parameter estimation. The analytical strategy followed a two-stage approach, beginning with the assessment of the measurement model, followed by the evaluation of the structural model.

In the first stage, the reliability and validity of the constructs were evaluated through indicator loadings, composite reliability (CR), average variance extracted (AVE), and discriminant validity using the Fornell–Larcker criterion. Once satisfactory measurement model fit was established, the structural model was assessed by analyzing the path coefficients, t-values, confidence intervals, and *p*-values generated via bootstrapping with 5,000 resamples. The model also incorporated interaction terms to test the moderating effects of peer support and examined indirect effects to validate the mediation and moderated mediation hypotheses. Multicollinearity was checked using variance inflation factors (VIF), and all values were within acceptable limits, confirming the stability of the model estimates.

To verify the robustness of the results, an additional covariance-based SEM (CB-SEM) was conducted using the maximum-likelihood estimation method. The CB-SEM model achieved satisfactory fit indices (χ^2^/df = 2.41, CFI = 0.948, TLI = 0.937, RMSEA = 0.052, SRMR = 0.046), all within recommended thresholds (Hair et al., 2019). The magnitude and direction of the path coefficients closely aligned with those obtained from the PLS-SEM analysis, confirming the stability and reliability of the findings.

#### Research findings

Table 1 presents the measurement items and their respective factor loadings for each latent construct used in the study. All item loadings exceed the recommended threshold of 0.70, except for one item under cultural adaptation (0.684) and one under student well-being (0.687), both of which are still within acceptable limits given their theoretical importance and the overall reliability of the constructs. These indicators were retained due to their strong theoretical relevance and their minimal influence on construct reliability and validity. When removed, Cronbach’s alpha (CA), composite reliability (CR), and average variance extracted (AVE) showed no substantive improvement, confirming the appropriateness of retention. The consistently high loadings across academic expectations, cultural adaptation, social integration, peer support, and student well-being indicate strong indicator reliability and support the convergent validity of the measurement model. These results confirm that the selected items effectively capture the intended constructs and are suitable for further structural analysis.

**Table 1:** Measurement scale items and their loadings.

Items	Loadings
Academic expectations	
I feel pressured to get high grades in all my subjects.	0.811
I worry about not meeting the academic expectations of my teachers.	0.802
The academic workload here is overwhelming.	0.784
I often fear academic failure due to unfamiliar standards.	0.738
Cultural adaptation	
I feel comfortable interacting with locals in daily situations.	0.763
I can adjust well to the academic culture of this country.	0.817
I understand the communication styles used here.	0.684
I am able to handle social customs appropriately.	0.716
I find adapting to this culture relatively easy.	0.847
I feel confident functioning in this new cultural setting.	0.713
Social integration	
I feel accepted by my classmates and peers.	0.790
I participate in social events and activities.	0.789
I have a sense of belonging at this university.	0.828
Peer support	
My peers help me when I am struggling.	0.827
I receive emotional support from my friends here.	0.781
I can depend on my classmates for academic help.	0.802
I often discuss my challenges with friends.	0.819
My peer group makes my life here easier.	0.728
Student well-being	
I feel mentally well and emotionally stable.	0.773
I am generally satisfied with my life here.	0.823
I feel positive about my day-to-day experiences.	0.687
I feel physically and emotionally healthy.	0.799
I maintain a good balance between academic and personal life.	0.800

Table 2 reports the reliability and validity assessment of the measurement model using CA, CR, AVE, and the Fornell–Larcker criterion. All constructs exceed the threshold for internal consistency, with Cronbach’s alpha values above 0.80 and CR values well above the recommended minimum of 0.70, indicating strong construct reliability.

**Table 2:** Reliability and validity (convergent and discriminant).

Fornell-Larcker						CA	CR	AVE
	AE	CA	SI	PS	SWB			
AE	0.784					0.877	0.899	0.615
CA	0.573	0.758				0.843	0.861	0.575
SI	0.285	0.682	0.802			0.900	0.921	0.644
PS	0.593	0.592	0.412	0.792		0.889	0.910	0.628
SWB	0.394	0.619	0.591	0.611	0.777	0.869	0.887	0.605

Convergent validity is supported as the AVE values for all constructs exceed 0.50, showing that each construct explains more than half of the variance in its indicators. Discriminant validity is confirmed using the Fornell–Larcker criterion, as the square root of the AVE (bold diagonal values) for each construct is greater than its correlations with other constructs in the matrix. This indicates that each construct is empirically distinct from the others. In addition to the Fornell–Larcker criterion, Heterotrait–Monotrait (HTMT) ratios were calculated to assess discriminant validity; all values were below the recommended threshold of 0.85 (Henseler et al., 2015), demonstrating that each construct is empirically distinct. The full HTMT matrix is presented in Appendix C. Collectively, these results validate the robustness of the measurement model and confirm that the constructs demonstrate both reliability and construct validity.

Table 3 presents the results of the collinearity diagnostics using variance inflation factor (VIF) values for all predictor constructs in the structural model. All VIF values are well below the commonly accepted threshold of 5.0, indicating the absence of multicollinearity issues among the predictors. The highest observed VIF is 2.22, which still falls within a safe range and suggests that the model estimates are stable and not adversely influenced by redundancy among the independent variables. These results confirm that collinearity does not pose a concern in the interpretation of path coefficients in the structural model.

**Table 3:** Collinearity assessment.

Variables	AE	CA	SI	PS	SWB
AE		1.19	1.98		1.44
CA					2.12
SI					1.49
PS		2.02	1.82		2.22
SWB					

Table 4 presents the results of the structural path model, confirming support for all hypothesized relationships in the proposed moderated mediation framework. The findings indicate that academic expectations have a significant negative effect on student well-being (B = −0.397, *p* = 0.000), suggesting that higher academic pressure is associated with lower levels of well-being among Chinese international students. Additionally, academic expectations negatively relate to both cultural adaptation (B = −0.412, *p* = 0.000) and social integration (B = −0.400, *p* = 0.006), indicating that students experiencing higher academic stress face greater difficulty adapting to the host culture and building social connections. In contrast, both cultural adaptation (B = 0.540, *p* = 0.000) and social integration (B = 0.612, *p* = 0.005) are positively associated with student well-being, demonstrating that those who are better adapted and socially integrated report higher well-being.

**Table 4:** Structural path model.

Relationships	B	SE	CIs	P	Decision
AE → SWB	−0.397	0.046	−0.512; −0.288	0.000	Supported
AE → CA	−0.412	0.049	−0.543; −0.300	0.000	Supported
AE → SI	−0.400	0.058	−0.525; −0.298	0.006	Supported
CA → SWB	0.540	0.055	0.410; 0.674	0.000	Supported
SI → SWB	0.612	0.061	0.498; 0.732	0.005	Supported
AE → CA → SWB	−0.333	0.048	−0.441; −0.229	0.000	Supported
AE → SI → SWB	−0.289	0.056	−0.383; −0.156	0.020	Supported
AE x PS → CA	0.388	0.044	0.267; 0.520	0.000	Supported
AE x PS → CA → SWB	0.350	0.052	0.223; 0.474	0.000	Supported
AE x PS → SI	0.410	0.048	0.300; 0.519	0.030	Supported
AE x PS → SI → SWB	0.398	0.045	0.289; 0.499	0.000	Supported

Standardized coefficients (β) are reported with standard errors (SE) in parentheses. All paths are significant at *p* < 0.05 unless otherwise indicated. Indirect and interaction effects are based on 5,000 bootstrap samples.

The mediation analysis confirms that both cultural adaptation and social integration significantly mediate the relationship between academic expectations and well-being. The indirect effect of academic expectations on well-being through cultural adaptation is significant and negative (B = −0.333, *p* = 0.000), as is the indirect path through social integration (B = −0.289, *p* = 0.020), confirming a parallel mediation structure. Furthermore, peer support significantly moderates the relationship between academic expectations and both mediators. The interaction effect of academic expectations and peer support on cultural adaptation is positive and significant (B = 0.388, *p* = 0.000), as is the interaction on social integration (B = 0.410, *p* = 0.030), indicating that peer support weakens the negative relationship between academic pressure and both adaptation and integration. The moderated mediation effects are also significant, as shown in the indirect paths through cultural adaptation (B = 0.350, *p* = 0.000) and social integration (B = 0.398, *p* = 0.000), revealing that peer support mitigates the negative association between academic stress and well-being via both mediators. These findings collectively validate the full moderated mediation model and highlight the critical buffering role of peer support in the academic and social adjustment process of international students.

Conditional indirect effects were computed to further examine the moderated mediation structure. Following Hayes (2018), the indirect effects of academic expectations on student well-being through cultural adaptation and social integration were estimated at three levels of peer support: low (−1 SD), medium (mean), and high (+1 SD). Bootstrapped 95% bias-corrected confidence intervals (5,000 resamples) were used to determine the significance of each conditional effect. The indirect effect through cultural adaptation was strongest at low peer support (*β* = −0.412, CI [−0.557, −0.287]), weaker at mean peer support (β = −0.276, CI [−0.415, −0.169]), and nonsignificant at high peer support (β = −0.101, CI [−0.246, 0.019]). A similar pattern was observed for the social integration path (low: β = −0.351, CI [−0.483, −0.217]; medium: β = −0.230, CI [−0.348, −0.127]; high: β = −0.092, CI [−0.210, 0.021]). These findings confirm that peer support effectively buffers the negative indirect effects of academic expectations on well-being through both adaptation and integration channels.

Effect size analysis further supports the practical importance of these relationships. The interaction term (AE × Peer Support) demonstrated a medium effect size (f^2^ = 0.12) for cultural adaptation and a small-to-medium effect (f^2^ = 0.08) for social integration. Predictive relevance values (q^2^ = 0.15 and 0.10, respectively) exceeded the minimum benchmark of 0.02, indicating that peer support meaningfully enhances the model’s explanatory power. Detailed conditional indirect effects are summarized in Appendix D.

#### Simple slope analysis

**Figure 2:**
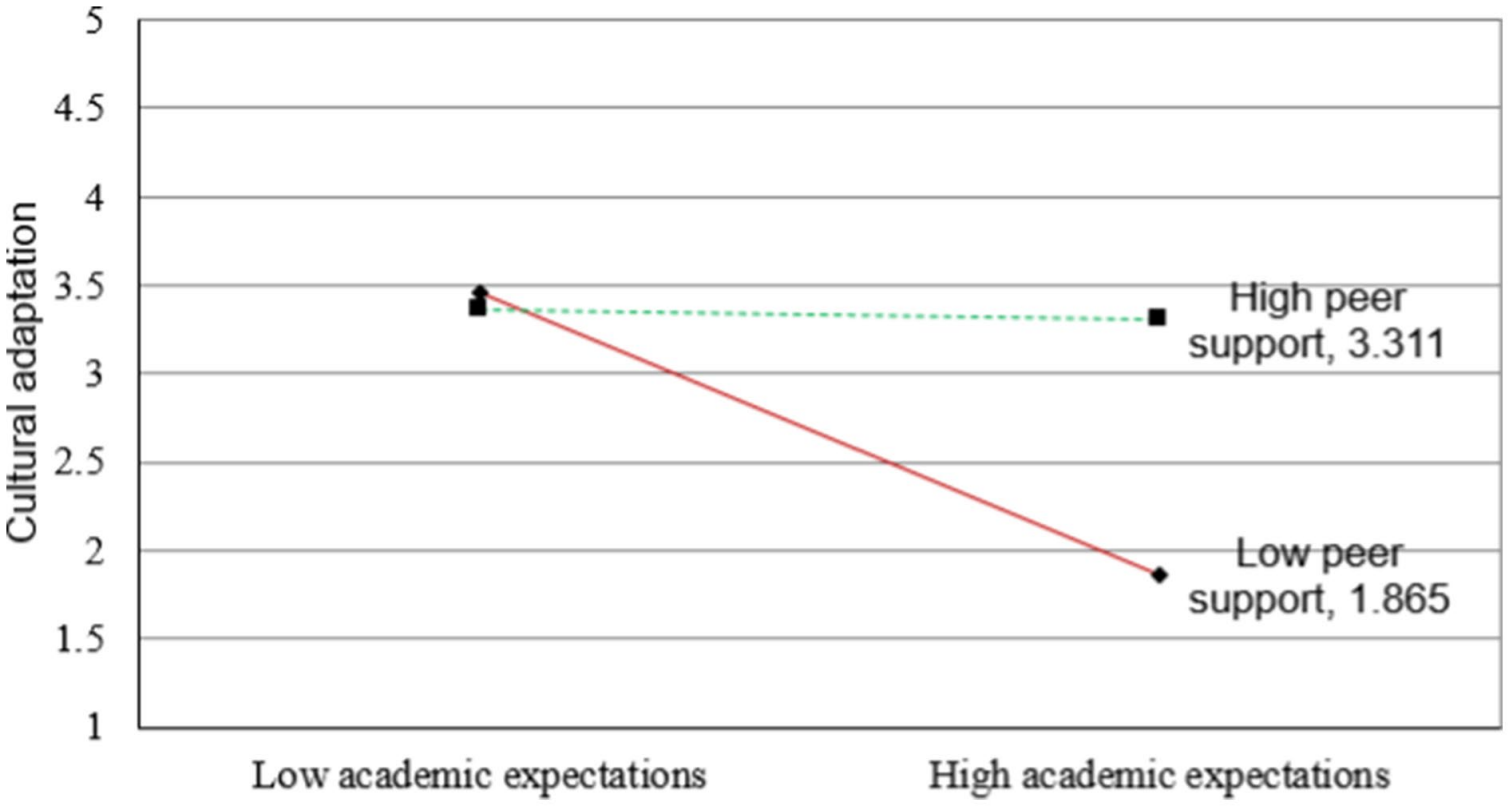
Interaction effect of peer support and academic expectations on cultural adaptation [each simple-slope plot displays lines for high peer support (+1 SD) and low peer support (−1 SD) conditions. The moderation effects were tested with 5,000 bootstraps, and corresponding 95% confidence intervals confirmed the significance of the slopes].

**Figure 3:**
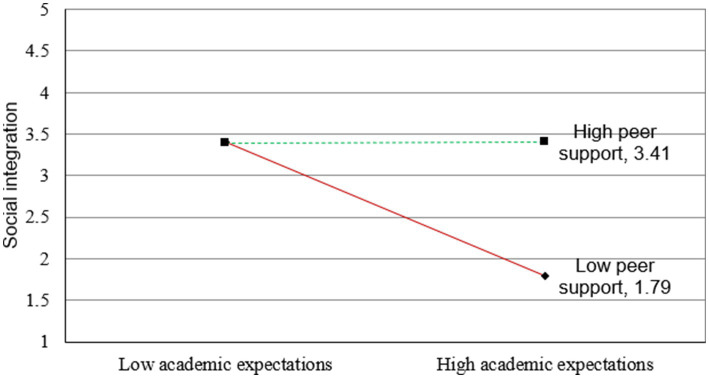
Interaction effect of peer support and academic expectations on social integration [each simple-slope plot displays lines for high peer support (+1 SD) and low peer support (−1 SD) conditions. The moderation effects were tested with 5,000 bootstraps, and corresponding 95% confidence intervals confirmed the significance of the slopes].

**Figure 4:**
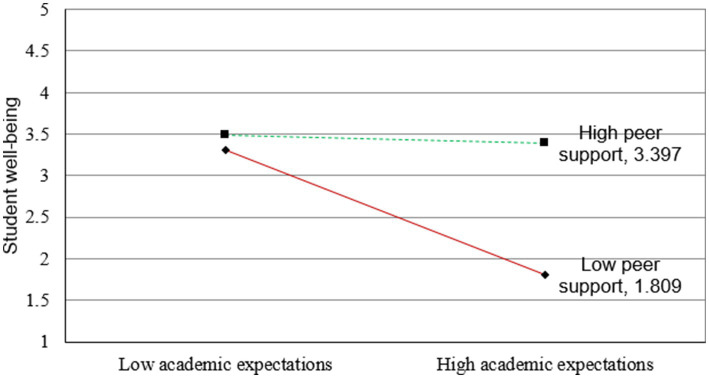
Conditional indirect effect of peer support and academic expectations on student well-being via cultural adaptation [each simple-slope plot displays lines for high peer support (+1 SD) and low peer support (−1 SD) conditions. The moderation effects were tested with 5,000 bootstraps, and corresponding 95% confidence intervals confirmed the significance of the slopes].

**Figure 5:**
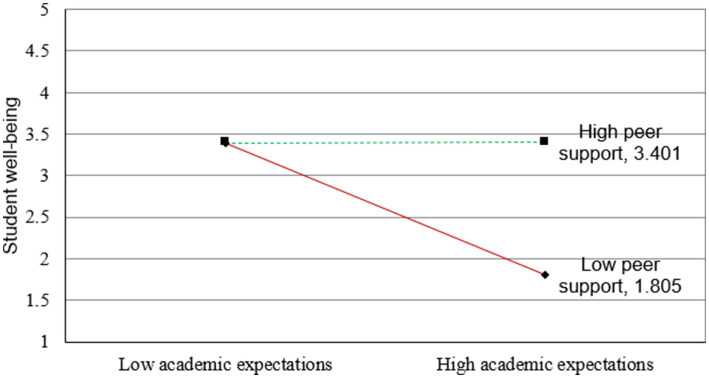
Conditional indirect effect of peer support and academic expectations on student well-being via social integration [each simple-slope plot displays lines for high peer support (+1 SD) and low peer support (−1 SD) conditions. The moderation effects were tested with 5,000 bootstraps, and corresponding 95% confidence intervals confirmed the significance of the slopes].

Figures 2–5 illustrate the results of the simple slope analyses conducted to examine the interaction effects of peer support and academic expectations on cultural adaptation, social integration, and student well-being through the two mediating pathways.

Figure 2 shows that the negative relationship between academic expectations and cultural adaptation is significantly moderated by peer support. Specifically, for students with high levels of peer support, the adverse relationship between academic expectations and cultural adaptation is notably weaker compared to those with low peer support. This indicates that peer support serves as a protective factor, enabling students to better adapt culturally even when facing high academic stress.

Similarly, Figure 3 demonstrates that peer support moderates the relationship between academic expectations and social integration. When peer support is high, the negative effect of academic stress on students’ social integration is considerably reduced. Conversely, under conditions of low peer support, academic expectations strongly hinder students’ ability to integrate socially. This reinforces the role of peer relationships in maintaining students’ social well-being under academic pressure.

Figures 4, 5 further extend this moderation effect to the indirect paths leading to student well-being. In Figure 4, the indirect negative effect of academic expectations on well-being through cultural adaptation is less pronounced when peer support is high, indicating a significant moderated mediation effect. Similarly, Figure 5 shows that peer support also attenuates the negative indirect relationship between academic expectations and well-being through social integration. In both cases, simple slope analyses confirm that the presence of strong peer support helps buffer the harmful effects of academic pressure, both by enhancing cultural and social adjustment and by preserving students’ overall well-being.

### Measurement invariance and subgroup analysis

To ensure the robustness and generalisability of the measurement model, the measurement invariance of composite models (MICOM) procedure was conducted following Henseler et al. (2015). The analysis assessed configural, compositional, and equality of mean and variance invariance across gender (male vs. female) and degree level (undergraduate vs. postgraduate) groups.

Results demonstrated configural and compositional invariance for all constructs, confirming that the factor structure and composite scores were equivalent across groups. Equality of composite means and variances was also established (ΔM < 0.01; ΔVar < 0.02), indicating that the constructs were measured equivalently across gender and degree level.

With measurement invariance confirmed, exploratory multi-group analyses (MGA) were performed to compare structural relationships between groups. Results revealed no significant path differences (*p* > 0.05) across gender or degree level, suggesting that the structural model operates similarly among male and female students and across educational stages.

These findings reinforce the generalisability of the proposed model and support its cross-group stability within the sampled population. Detailed MICOM and multi-group results are provided in Appendix E.

## Discussion

To date, the present research is one of the first empirical efforts to examine how academic expectations relate to student well-being through the mediating mechanisms of cultural adaptation and social integration, and the moderating role of peer support among Chinese international students. All five hypotheses proposed in the conceptual framework were statistically supported, lending robust evidence to the moderated mediation model based on Acculturative Stress Theory (Berry, 1997). This study not only extends theoretical understanding of student adaptation in cross-cultural contexts but also offers practical insights into institutional interventions for student support. The results are discussed in detail below.

The first hypothesis (H1) was supported, showing that academic expectations significantly and negatively relate to student well-being. This finding aligns with previous literature demonstrating that heightened academic pressure contributes to psychological distress, anxiety, and decreased life satisfaction among international students (Granieri et al., 2021; Kristensen et al., 2023). The theoretical underpinning is consistent with Acculturative Stress Theory, which suggests that academic demands function as environmental stressors that can undermine emotional well-being when coping resources are inadequate (Berry, 1997). These results further support the argument that international students often experience intensified academic stress due to unfamiliar instructional methods, language difficulties, and perceived performance expectations (Ripani, 2024). Thus, the negative association between academic expectations and well-being is both theoretically grounded and empirically validated.

Second, this study confirmed the mediating role of cultural adaptation in the relationship between academic expectations and student well-being (H2a). The findings revealed that students who successfully adapt to the academic and cultural environment of the host country are better able to cope with academic stress, thereby preserving their psychological well-being. This result is consistent with previous studies that show well-adapted students experience greater emotional stability, academic confidence, and resilience under pressure (Campos et al., 2022; Stockinger et al., 2021). The underlying mechanism is rooted in the cognitive and behavioral adjustments that facilitate the interpretation of stressful academic demands as manageable challenges rather than threats (Besser et al., 2022; Soares et al., 2021; Wang et al., 2021). Therefore, this study empirically establishes cultural adaptation as a crucial psychological buffer in the academic stress process.

The third hypothesis (H2b) was also supported, indicating that social integration significantly mediates the association between academic expectations and student well-being. Students with higher levels of social connectedness and campus involvement reported better psychological outcomes, even in the presence of academic pressure. These findings echo previous work that highlights the importance of social belonging and peer interaction in reducing emotional distress and enhancing satisfaction (Bradley et al., 2021; Edwards et al., 2022). Within the framework of acculturative stress, social integration operates as an interpersonal coping mechanism that facilitates emotional regulation and academic persistence (Li et al., 2025; Permatasari et al., 2021). Thus, similar to cultural adaptation, social integration functions as a mediating variable that transforms academic stress into a more manageable experience through community-based support and emotional safety.

Hypotheses H3 and H4 tested the moderating role of peer support on the relationships between academic expectations and the two mediators: cultural adaptation and social integration. Both hypotheses were supported. The findings reveal that peer support significantly buffers the negative relationship between academic expectations and both adaptation and integration processes. These results reinforce the buffering hypothesis of social support (Fraser et al., 2024), suggesting that students who perceive high levels of peer support are better equipped to interpret academic stress as less overwhelming, thereby facilitating smoother cultural adaptation and stronger social engagement. This empirical evidence is in line with earlier studies, which found that peer interactions enhance emotional stability, encourage cultural learning, and promote campus inclusion among international students (Besser et al., 2022; Stockinger et al., 2021). Therefore, the presence of peer support serves as a contextual moderator that shapes how academic expectations translate into adaptive or maladaptive adjustment outcomes.

Finally, the findings supported the moderated mediation hypotheses (H5a and H5b), demonstrating that peer support not only moderates the direct effects of academic expectations on adaptation and integration, but also the indirect effects on student well-being through both pathways. Specifically, the indirect negative effects of academic expectations on well-being were significantly weaker at higher levels of peer support. This finding integrates the theoretical insights from Acculturative Stress Theory and the buffering hypothesis to explain how supportive peer environments facilitate psychological resilience even in high-pressure academic settings (Lashari et al., 2023; Su et al., 2021). Prior studies have emphasized the importance of peer-based interventions in improving cross-cultural adaptation, but few have empirically validated their role in conditional indirect pathways (Shahid and Shabbir, 2021; Zhao and Schartner, 2024). The current study contributes to filling this gap by confirming the protective function of peer networks in the broader framework of student well-being and adjustment. Future research might also examine how peer support interacts with institutional or faculty support in buffering acculturative stress, as the combined influence of interpersonal and structural resources may further enhance international students’ adjustment and well-being.

In sum, the empirical results of this study reinforce the complexity of international student adaptation by demonstrating that academic expectations do not operate in isolation but are filtered through psychological, cultural, and interpersonal processes. While many previous studies support the direct impact of academic stress on mental health, the present findings suggest that this relationship is influenced by deeper mechanisms involving cultural learning, social bonding, and peer support. The integration of moderated mediation analysis offers a nuanced understanding of how stress is processed differently based on social context, thereby extending both theory and practice in the field of international higher education.

## Conclusion

The objective of the present research was to examine how academic expectations affect the well-being of Chinese international students, with a specific focus on the mediating roles of cultural adaptation and social integration, as well as the moderating and moderated-mediating effects of peer support. Using cross-sectional survey data and structural equation modeling, the empirical findings demonstrated that academic expectations have a significant negative association with student well-being. In addition, both cultural adaptation and social integration were found to mediate this relationship, highlighting their role as key psychological and social pathways in the academic adjustment process. Furthermore, the study confirmed that peer support significantly moderates the relationships between academic expectations and both mediators, and also buffers the overall indirect effects on well-being.

This study offers both theoretical and practical contributions. Theoretically, it extends the application of Acculturative Stress Theory by integrating mediation and moderation mechanisms within a conditional process model, thereby enriching the understanding of how academic stress influences psychological outcomes among international students. The inclusion of peer support as a contextual moderator advances the literature on student adaptation and support systems in higher education. Practically, the findings emphasize the importance of developing targeted institutional strategies that support cultural and social integration for international students. Universities should invest in peer mentoring programs, intercultural engagement initiatives, and academic orientation workshops to facilitate smoother transitions and reduce stress-related challenges. At a broader level, this research provides a foundation for future exploration of culturally embedded psychological processes, emphasizing the need for inclusive educational policies that prioritize international student well-being in increasingly globalized academic environments.

### Practical implications

The findings of this study offer several practical implications for higher education institutions, student affairs professionals, and policymakers concerned with the well-being of international students. The path coefficient from academic expectations to well-being (*β* = −0.397) confirms that academic expectations constitute a critical source of stress, particularly when these expectations are unfamiliar, rigid, or excessively high. Therefore, academic advisors and faculty members must provide clear, culturally sensitive guidance on academic standards, performance expectations, and learning outcomes. Orientation programs and academic workshops specifically designed for international students can help align their expectations with institutional realities, thereby reducing stress and enhancing academic confidence.

Second, the results highlight the importance of cultural adaptation and social integration as psychological and social buffers that mediate the association between academic pressure and well-being. Both mediators showed large positive effects on well-being (β = 0.540 and β = 0.612, respectively), indicating that strengthening these areas can produce substantial improvements in students’ adjustment. Institutions should actively facilitate cultural adaptation by offering intercultural training sessions, language support programs, and culturally inclusive pedagogical practices. At the same time, fostering a socially engaging campus environment is essential. Organizing peer-based activities, inclusive student organizations, and cross-cultural events can significantly enhance students’ sense of belonging and social connectedness, which in turn contributes to better psychological outcomes.

Third, the moderating role of peer support suggests that interpersonal relationships play a pivotal role in shaping how students manage academic and cultural challenges. Moderation analyses revealed medium effect sizes (f^2^ = 0.12 for cultural adaptation; f^2^ = 0.08 for social integration) and predictive relevance values (q^2^ = 0.15 and 0.10), indicating that peer support meaningfully buffers the negative association between academic pressure and these mediators. Universities should therefore implement peer-mentoring schemes where experienced students support newcomers in navigating academic systems, cultural norms, and campus life. These programs can help create a psychologically safe and supportive environment, particularly for students who are at risk of isolation or stress. Encouraging informal social spaces, group-based coursework, and collaborative learning environments can further strengthen peer bonds.

Finally, institutional policies must be inclusive and responsive to the unique challenges faced by international students. Administrative bodies should regularly assess student well-being, track engagement in support services, and allocate resources to promote intercultural engagement and psychosocial support. Because the observed effects are moderate to large in magnitude, even incremental improvements in peer engagement and adaptation programs are likely to yield practically meaningful gains in student well-being. By addressing both the structural and interpersonal dimensions of student life, institutions can play a critical role in enhancing the resilience, adjustment, and overall well-being of their international student communities.

### Limitations and avenues for future studies

Despite its contributions, this study is not without limitations. First, the cross-sectional nature of the research design limits the ability to make causal inferences about the relationships among academic expectations, cultural adaptation, social integration, peer support, and student well-being. Although the hypothesized paths are theoretically grounded and empirically supported, future studies should adopt longitudinal or experimental designs to assess changes over time and establish temporal precedence. Second, data were collected solely from Chinese international students, which limits the generalizability of the findings to other nationalities or cultural groups. Future research should replicate the model across more diverse international student populations to test for cultural invariance and enhance external validity.

Third, the reliance on self-reported data may introduce potential biases, such as social desirability or common method variance, despite efforts to minimize these risks through procedural and statistical controls. Future studies could incorporate multi-source data, including peer or faculty assessments, or objective academic performance indicators to strengthen the robustness of the findings. Fourth, while the current study focuses on cultural adaptation and social integration as mediators, and peer support as a moderator, other relevant psychological variables—such as resilience, emotional regulation, or institutional support—could provide additional explanatory power. Future research may benefit from exploring these alternative or complementary mechanisms within a broader conceptual framework.

Lastly, the study was conducted within specific university contexts in a limited geographic region. Institutional policies, academic cultures, and support services may vary significantly across countries and institutions, potentially influencing student adjustment processes. Future research should consider multi-institutional or cross-national comparisons to explore how contextual factors interact with individual-level variables. Expanding the scope of inquiry in this manner will contribute to a more comprehensive understanding of international student well-being in an increasingly globalized higher education landscape. Despite these limitations, this study offers a valuable foundation for future cross-cultural work on student well-being.

## Data Availability

The raw data supporting the conclusions of this article will be made available by the authors, without undue reservation.
