# Supplementary material for: Navigating academic expectations and social integration: a moderated mediation analysis of cultural adaptation, peer support, and well-being among Chinese international students
Source: Front Psychol. 2025 Nov 21;16:1633773. doi: 10.3389/fpsyg.2025.1633773 (PMC12679276; doi:10.3389/fpsyg.2025.1633773)
Supplement: Supplementary file 1 [file Data_Sheet_1.docx]

| Appendix A. Demographics | | | |
| --- | --- | --- | --- |
| Variable | Category | n | % |
| Gender | Male | 177 | 38.8 |
|  | Female | 279 | 61.2 |
| Age Group (years) | 18–22 | 239 | 52.4 |
|  | 23–25 | 96 | 21.1 |
|  | 26 and above | 121 | 26.5 |
| Education Level | Undergraduate | 296 | 64.9 |
|  | Postgraduate | 160 | 35.1 |
| Duration of Stay | Less than 6 months | 87 | 19.1 |
|  | 6–12 months | 192 | 42.1 |
|  | More than 1 year | 177 | 38.8 |
| Language Proficiency | Low | 80 | 17.6 |
|  | Moderate | 231 | 50.7 |
|  | High | 145 | 31.7 |

| Appendix B. Common method bias diagnostics | | |
| --- | --- | --- |
| Test Type | Statistic / Range | Interpretation |
| Harman’s single-factor variance | 28.4% | < 50% → No major CMB |
| CFA single-factor model fit | χ²/df = 7.12, CFI = 0.62, TLI = 0.58, RMSEA = 0.11, SRMR = 0.09 | Poor fit → No single-factor dominance |
| Full Collinearity VIFs | 1.57–2.22 | < 3.3 → Acceptable |
| Marker-variable correlation | Nonsignificant | Confirms minimal CMB |
| Note. Multiple diagnostic tests collectively confirm that common method bias is not a serious threat to the validity of the study’s findings. | | |

| Appendix C. Heterotrait-monotrait (HTMT) ratio | | | |
| --- | --- | --- | --- |
| Construct Pair | HTMT Value | Threshold | Decision |
| Academic expectations – Cultural adaptation | 0.641 | ≤ 0.85 | Accepted |
| Academic expectations – Social integration | 0.713 | ≤ 0.85 | Accepted |
| Academic expectations – Peer support | 0.590 | ≤ 0.85 | Accepted |
| Cultural adaptation – Social integration | 0.773 | ≤ 0.85 | Accepted |
| Cultural adaptation – Peer support | 0.622 | ≤ 0.85 | Accepted |
| Social integration – Peer support | 0.684 | ≤ 0.85 | Accepted |
| Social integration – Well-being | 0.730 | ≤ 0.85 | Accepted |
| Cultural adaptation – Well-being | 0.706 | ≤ 0.85 | Accepted |
| Peer support – Well-being | 0.666 | ≤ 0.85 | Accepted |

| Appendix D. Conditional indirect effects and effect sizes | | | | | | |
| --- | --- | --- | --- | --- | --- | --- |
| Mediating Path | Peer Support Level | Indirect Effect (β) | 95% BCa CI [Lower, Upper] | f² | q² | Significance |
| AE → CA → WB | Low (–1 SD) | –0.412 | [–0.557, –0.287] | 0.12 | 0.15 | Yes |
| AE → CA → WB | Medium | –0.276 | [–0.415, –0.169] | – | – | Yes |
| AE → CA → WB | High (+1 SD) | –0.101 | [–0.246, 0.019] | – | – | No |
| AE → SI → WB | Low (–1 SD) | –0.351 | [–0.483, –0.217] | 0.08 | 0.10 | Yes |
| AE → SI → WB | Medium | –0.230 | [–0.348, –0.127] | – | – | Yes |
| AE → SI → WB | High (+1 SD) | –0.092 | [–0.210, 0.021] | – | – | No |

| Appendix E. MICOM and multigroup analysis summary | | | | | |
| --- | --- | --- | --- | --- | --- |
| Group Split | Configural Invariance | Compositional Invariance | Mean/Variance Invariance | MGA Result (Path Differences) | Interpretation |
| Gender (Male vs. Female) | Supported | Supported | Supported | p > 0.05 | No significant differences |
| Degree Level (Undergraduate vs. Postgraduate) | Supported | Supported | Supported | p > 0.05 | No significant differences |
